# Nymphomania in a Mare Cured by Chloral

**Published:** 1877-11

**Authors:** 


NYMPHOMANIA IN A MARE CURED BY CHLORAL.—A mare that was affected with nyphomania to such a degree that she was useless for any sort of work, was recently brought to the veterinary surgeon, Ritz, of Berlin. The following were the chief symptoms: wildness, general emaciation, eyes haggard, conjunctiva injected, pupils dilated, prehension of food irregular, mastication slow and occasionally intermittent. To calm the genital excitement, Ritz ordered twenty grammes (5 drachms) of chloral to be administered, dissolved in the drink. After a few days, the nymphomania disappeared, and the mare gradually recovered, and gained flesh rapidly.

